# MUC5AC Expression in Various Tumor Types and Nonneoplastic Tissue: A Tissue Microarray Study on 10 399 Tissue Samples

**DOI:** 10.1177/15330338211043328

**Published:** 2021-09-21

**Authors:** Sebastian Dwertmann Rico, Moritz Mahnken, Franziska Büscheck, David Dum, Andreas M. Luebke, Martina Kluth, Claudia Hube-Magg, Andrea Hinsch, Doris Höflmayer, Christina Möller-Koop, Christoph Fraune, Katharina Möller, Anne Menz, Christian Bernreuther, Frank Jacobsen, Patrick Lebok, Till S. Clauditz, Guido Sauter, Ria Uhlig, Waldemar Wilczak, Ronald Simon, Stefan Steurer, Sarah Minner, Eike Burandt, Till Krech, Andreas H. Marx

**Affiliations:** 1 Institute of Pathology, University Medical Center Hamburg-Eppendorf, Hamburg, Germany; 2 Institute of Pathology, Clinical Center Osnabrueck, Osnabrueck, Germany; 3 Academic Hospital Fuerth, Fuerth, Germany

**Keywords:** MUC5AC, multitumor tissue microarray, immunohistochemistry

## Abstract

**Background:** Mucin 5AC (MUC5AC) belongs to the glycoprotein family of secreted gel-forming mucins and is physiologically expressed in some epithelial cells. Studies have shown that MUC5AC is also expressed in several cancer types suggesting a potential utility for the distinction of tumor types and subtypes. **Methods:** To systematically determine MUC5AC expression in normal and cancerous tissues, a tissue microarray containing 10 399 samples from 111 different tumor types and subtypes as well as 608 samples of 76 different normal tissue types was analyzed by immunohistochemistry. **Results:** MUC5AC was expressed in normal mucus-producing cells of various organs. At least weak MUC5AC positivity was seen in 44 of 111 (40%) tumor entities. Of these 44 tumor entities, 28 included also tumors with strong positivity. MUC5AC immunostaining was most commonly seen in esophageal adenocarcinoma (72%), colon adenoma (62%), ductal adenocarcinoma of the pancreas (64%), mucinous carcinoma of the ovary (46%), diffuse gastric adenocarcinoma (44%), pancreatic ampullary adenocarcinoma (41%), intestinal gastric adenocarcinoma (39%), and bronchioloalveolar carcinoma (33%). Clinically relevant tumors with complete or almost complete absence of MUC5AC staining included small cell carcinoma of the lung (0% of 17), clear cell renal cell carcinoma (0% of 507), papillary thyroid carcinoma (0% of 359), breast cancer (2% of 1097), prostate cancer (2% of 228), soft tissue tumors (0.1% of 968), and hematological neoplasias (0% of 111). **Conclusion:** The highly standardized analysis of a broad range of cancers identified a ranking order of tumors according to their relative prevalence of MUC5AC expression.

## Introduction

Mucin 5AC (MUC5AC) belongs to the subset of 5 secreted gel-forming mucins (MUC2, -5B, -5AC, -6, and -19) which are encoded by a gene cluster located at chromosome 11p15. MUC5AC is a glycoprotein with multiple cysteine-rich domains in both N- and C-terminal regions that are responsible for the formation of polymers, a critical feature for gel forming.^1^ The mucin layer protects the epithelial surfaces from chemical and mechanical damage as well as microbial pathogens, which are bound and subsequently removed by the mucociliary system.^2,3^ MUC5AC is normally expressed in epithelia of the upper and lower respiratory tract, stomach, and endocervix ,^4–7^ but it can be aberrantly expressed in a variety of cancers and their precursor lesions, including tumors arising from epithelia that are physiologically MUC5AC negative.^8–14^ There is growing evidence that MUC5AC expression in cancer cells may actively contribute to tumor aggressiveness. For example, it has been suggested that MUC5AC may interact with integrin β4 to facilitate lung cancer metastasis,^15^ enhance colorectal cancer tumorigenesis by deregulation of p53 and ß-catenin^16^ and repress apoptosis and cadherin-dependent cell adhesion in pancreatic cancer cells.^17,18^

Several previous studies have, moreover, shown that MUC5AC immunostaining could offer additional diagnostic information in various tumor types. For example, aberrant MUC5AC expression has been found in intraductal papillary mucinous neoplasia and pancreatic cancers, suggesting that MUC5AC can aid in assuring the diagnosis of pancreatic carcinomas,^19^ classifications of gastric polyps and Barrett metaplasia,^20^ or as a diagnostic tool in the dermapathology.^21^ It is also suggested that MUC5AC expression is helpful to distinguish between sessile serrated adenomas/polyps and hyperplastic polyps.^22^ Studies analyzing MUC5AC expression in cancers by immunohistochemistry (IHC) have described highly variable data. For example, the described frequency of positive MUC5AC immunostaining ranged from 29% to 100% in nonsmall-cell lung cancer,^23,24^ 15% to 100% in lung adenocarcinoma,^13,25^ 25% to 85% in gastric cancer,^26,27^ 0% to 100% in ovarian carcinoma,^9,28^ 28% to 80% of the ampulla Vater,^29,30^ or 14% to 54% in cervical carcinomas.^12,31^ Technical factors, such as staining protocols and antibodies used, different definitions of thresholds to determine positivity, as well as possible selection bias with respect to the analyzed tumors may have caused these discrepancies. To better understand the relative importance of MUC5AC expression in different cancer types and normal tissues, a comprehensive study analyzing many cancerous and noncancerous tissues under highly standardized conditions is desirable.

This study was thus designed to collect comparable data on the rate of MUC5AC expression in a broad range of different tissues. For this purpose, more than 10 000 tissue samples from 111 different tumor types and subtypes, and 76 nonneoplastic tissues were evaluated by IHC in a tissue microarray (TMA) format.

## Material and Methods

### Tissue Microarrays

To study MUC5AC expression in normal and neoplastic human tissues, we used a preexisting TMA containing 10 399 primary tumors from 111 tumor types and subtypes as well as 608 samples of 76 different normal tissues. All samples were derived from the archives of the Institute of Pathology, University Hospital of Hamburg, Germany, the Institute of Pathology, Clinical Center Osnabrueck, Germany, and the Department of Pathology, Academic Hospital Fuerth, Germany. Tissues were fixed in 4% buffered formalin and then embedded in paraffin. TMA tissue spot diameter was 0.6 mm. The usage of archived diagnostic leftover tissues for manufacturing of TMAs and their analysis for research purposes as well as patient data analysis has been approved by local laws (HmbKHG, §12) and by the local ethics committee (Ethics commission Hamburg, WF-049/09). All work has been carried out in compliance with the Helsinki Declaration.

### Immunohistochemistry

Freshly cut TMA sections were immunostained on 1 day and in 1 experiment. Slides were deparaffinized and exposed to heat-induced antigen retrieval for 5 min in an autoclave at 121 °C in pH 7.8 Dako Target Retrieval Solution buffer (Dako). A primary antibody specific against MUC5AC protein (mouse monoclonal, MSVA-109, MS Validated Antibodies) was applied at 37 °C for 60 min at a dilution of 1:200. Bound antibody was then visualized using the EnVision Kit (Dako) according to the manufacturer's directions. For tumor tissues, the percentage of positive neoplastic cells was estimated, and the staining intensity was semiquantitatively recorded (0, 1+, 2+, 3+). For statistical analyses, the staining results were categorized into 4 groups. Tumors without any staining were considered to be negative. Tumors with 1+ staining intensity in ≤70% of cells or 2+ intensity in ≤30% of cells were considered weakly positive. Tumors with 1+ staining intensity in >70% of cells, 2+ intensity in 31% to 70%, or 3+ intensity in ≤30% were considered moderately positive. Tumors with 2+ intensity in >70% or 3+ intensity in >30% of cells were considered strongly positive.

### Statistics

No statistical analysis was performed in this study. The list of tumor types and the fraction of MUC5AC positive samples per tumor type was generated using JMP 14 software (SAS Institute Inc.).

## Results

### Technical Issues

A total of 8028 (77.2%) of 10 399 tumor samples and >500 normal samples were interpretable in our TMA analysis. Noninterpretable samples demonstrated lack of unequivocal tumor cells.

### MUC5AC in Normal Tissues

**Figure 1.**
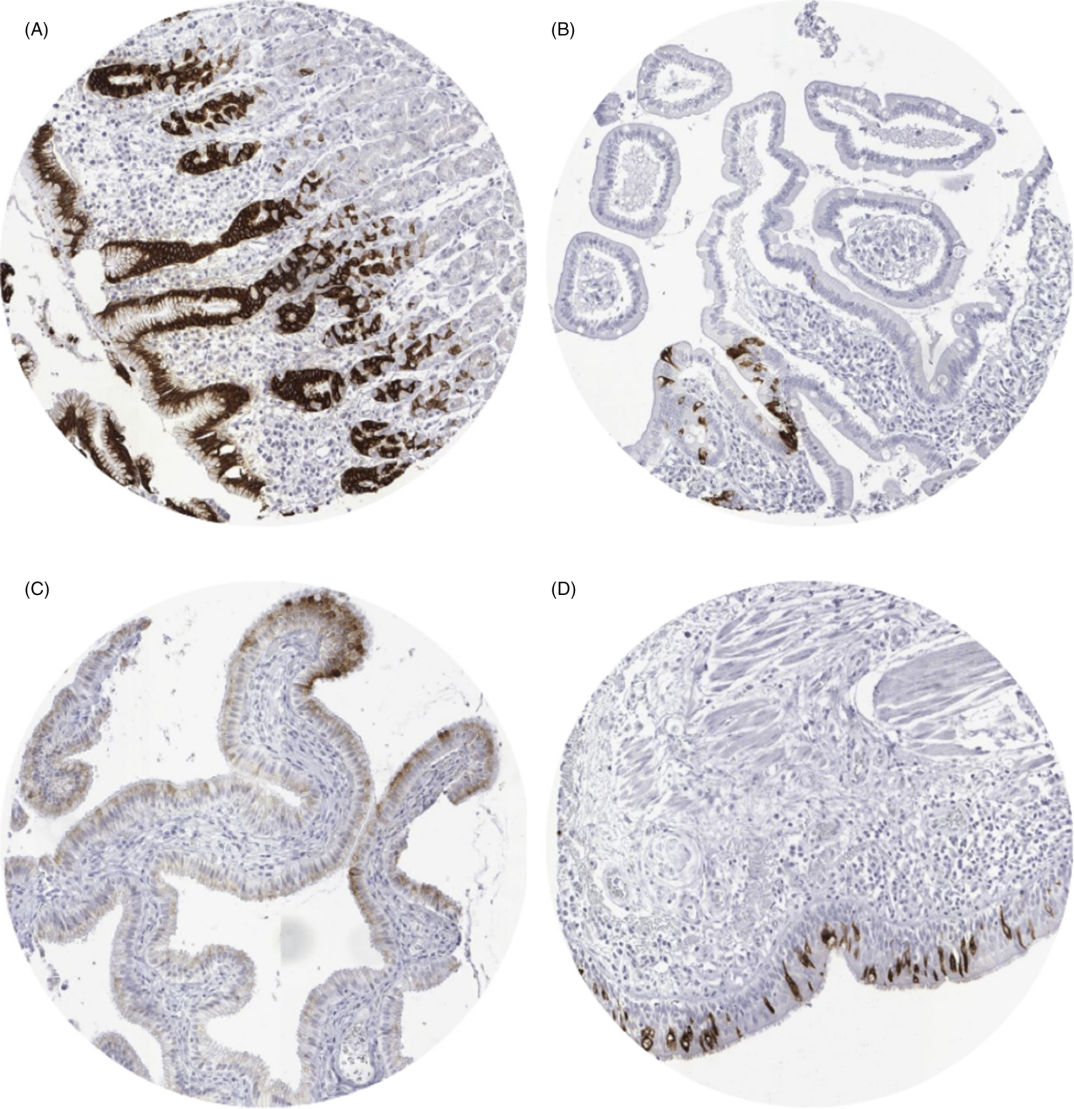
Mucin 5AC (MUC5AC) staining in normal tissues. (A) Stomach, corpus, (B) duodenum (C) gallblader, (D) bronchial mucosa.

A strong (3+) cytoplasmic MUC5AC staining was found in 100% of the columnar cells of the stomach surface epithelium (Figure 1A), a small fraction of surface epithelial cells of the duodenum (Figure 1B), small intestine, appendix, and colon, columnar surface cells of the transitional epithelium of the anal canal, columnar cells of the gallbladder surface epithelium (2+, Figure 1C), and in goblet cells of the bronchial system (Figure 1D) and the paranasal sinuses (3+). In addition, moderate to strong MUC5AC staining was seen in normal appearing endocervical glands from 2 of 7 donors (2+). MUC5AC staining was absent in aorta/intima, aorta/media, heart (left ventricle), skeletal muscle, skeletal muscle/tongue, myometrium, muscular wall of the gastrointestinal (GI)-tract (appendix, esophagus, stomach, ileum, and colon descendens), muscular wall of the renal pelvis and bladder, glans of the penis, ovarian stroma, keratinocytes of the epidermis, sebaceous glands, lip, oral cavity, surface of the tonsil, epidermis of the anal canal, mucosa and submucosa of the esophagus, squamous epithelium of the ectocervix, urothelial cells of the bladder and renal pelvis, lamina propria of the bladder and renal pelvis, amnion/chorion, lymph nodes, spleen, thymus, tonsil, lamina propria of the duodenum, ileum, colon descendens, rectum, appendix and gallbladder, placental trophoblastic cells (cytotrophoblast and syncytiotrophoblast), decidua, gastric epithelial cells, enterocytes of the small and large intestine including appendix, hepatocytes, Kupffer cells, bile duct epithelium of the liver, pancreas, renal cortex and medulla, prostate, epididymis, mucosa and submucosa of the lung bronchi (except globlet cells), mucinous and/or serous epithelium as well as ductal cells of the salivatory glands (parotis, glandular submandibularis and glandular sublingualis), thyroid, sertoli cells, leydig cells, testicular germ cells, submucosal bronchial glands, bronchus epithelium, pneumocytes, epithelium of the paranasal sinus (except goblet cells), glandular and ductal epithelium of the breast, endometrium, fallopian tube, ovary (corpus luteum and follicular cyst), parathyroid gland, cortical cells of the adrenal gland, adrenal medullary cells, neuronal and glial cells of the cerebrum and cerebellum, cells of the neurohypophysis and adenohypophysis.

### MUC5AC in Tumor Tissues

**Figure 2.**
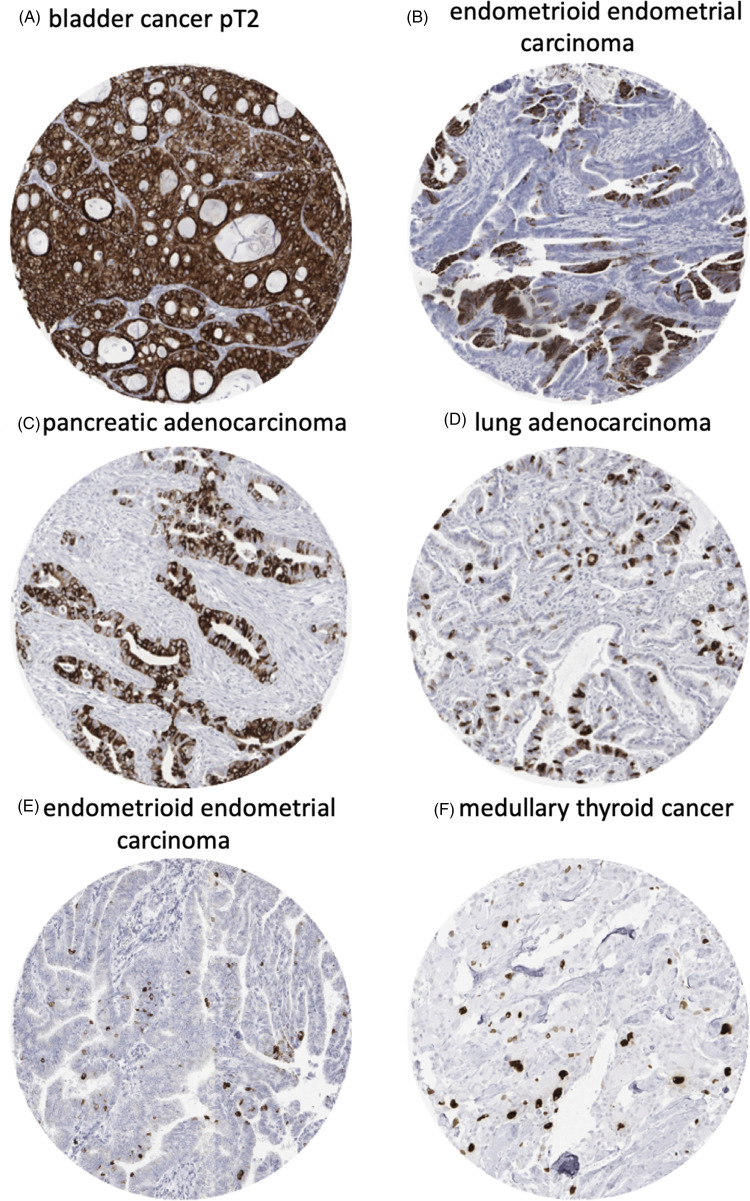
Mucin 5AC (MUC5AC) staining in different tumor types and subtypes. (A) Bladder cancer pT2, (B) endometrioid endometrial carcinoma, (C) pancreatic adenocarcinoma, (D) lung adenocarcinoma, (E) endometrioid endometrial carcinoma, (F) medullary thyroid cancer.

**Figure 3.**
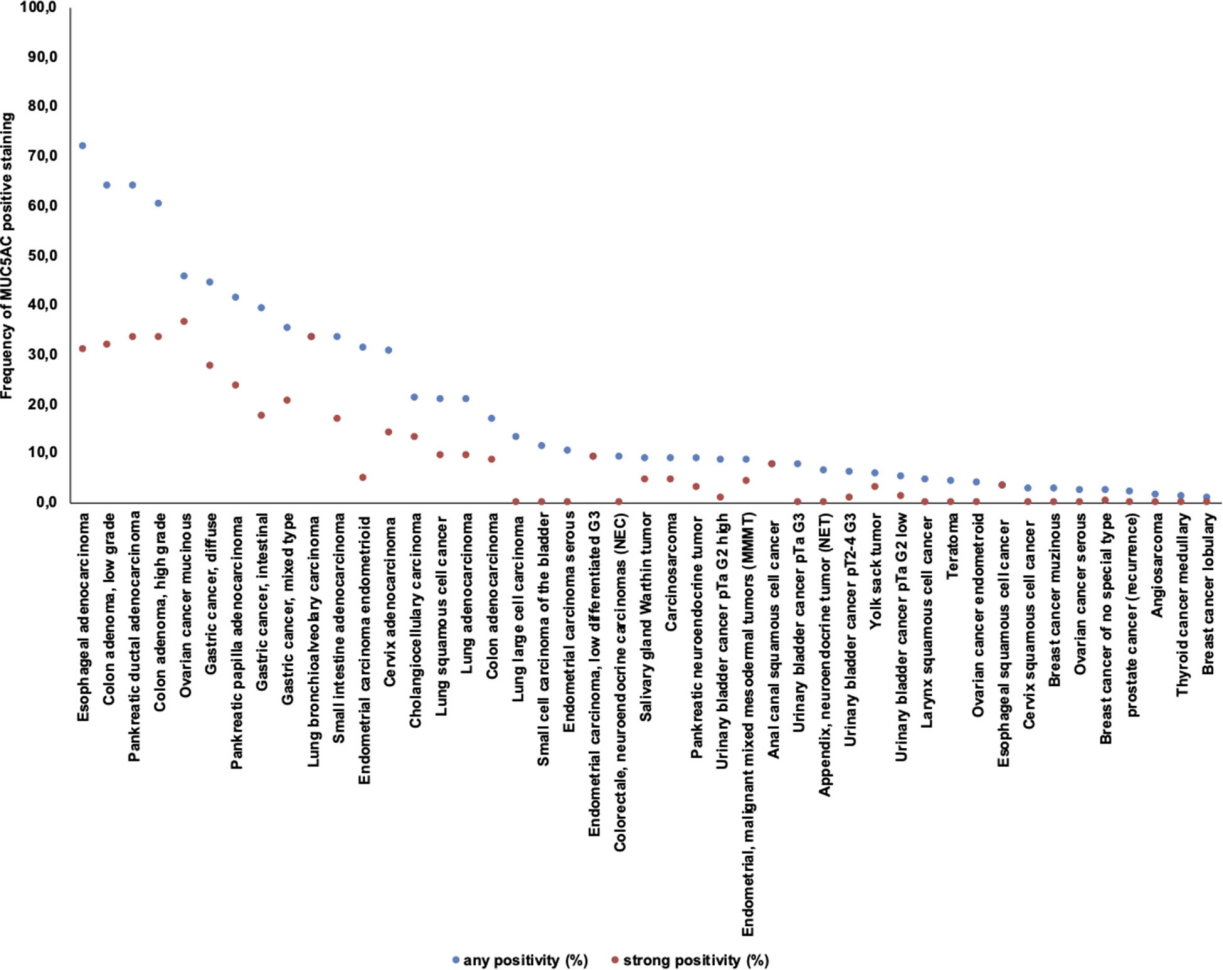
Ranking order of Mucin 5AC (MUC5AC) immunostaining in tumors. Both the frequency of positive cases (blue dots) and the frequency of strongly positive cases (orange dots) are shown.

MUC5AC immunostaining was cytoplasmic and sometimes showed a tendency towards particular strong staining at the apical pole of tumor cells. Some tumors showed intense diffuse staining involving all cells while others showed patchy staining of either group of cells or even scattered individual cells. Representative images are given in Figure 2. In total, 540 (6.7%) of 8028 analyzable tumors showed positive immunostaining for MUC5AC (Table 1). MUC5AC immunostaining was considered weak in 152 (1.9%), moderate in 185 (2.3%), and strong in 203 (2.5%) of tumors. Overall, 44 of 111 (39.6%) tumor categories showed a detectable MUC5AC expression with 28 (25.2%) categories showing at least in a small proportion strong positivity. The categories with the highest rate of positive staining (33%-72%) included adenocarcinomas of various types, primarily from the GI tract, the female genital tract, and the lung. These tumor types also showed the highest fractions of tumors with strong positivity (17%-31%). Important tumor types with low or absent MUC5AC immunostaining included sarcomas, lymphomas, endocrine tumors, renal cancer, breast cancer, prostate cancer, and various skin tumors. A graphical representation of ranking order of MUC5AC positive and strongly positive cancers is given in Figure 3.

**Table 1.** MUC5AC Immunostaining in Human Tumors.

	Tumor entity	*n* on TMA	MUC5AC immunostaining					
			*n* analyzable	Negative (%)	Weak (%)	Moderate (%)	Strong (%)	Positive (%)
Tumors of the skin	Pilomatrixoma	35	26	100.0	0.0	0.0	0.0	0.0
	Basal cell carcinoma	48	47	100.0	0.0	0.0	0.0	0.0
	Benign nevus	29	22	100.0	0.0	0.0	0.0	0.0
	Squamous cell carcinoma of the skin	50	44	100.0	0.0	0.0	0.0	0.0
	Malignant melanoma	48	44	100.0	0.0	0.0	0.0	0.0
	Merkel cell carcinoma	46	42	100.0	0.0	0.0	0.0	0.0
Tumors of the head and neck	Squamous cell carcinoma of the larynx	50	43	95.3	0.0	4.7	0.0	4.7
	Oral squamous cell carcinoma (floor of the mouth)	50	42	100.0	0.0	0.0	0.0	0.0
	Pleomorphic adenoma of the parotid gland	50	42	100.0	0.0	0.0	0.0	0.0
	Warthin tumor of the parotid gland	49	45	91.1	2.2	2.2	4.4	8.9
	Basal cell adenoma of the salivary gland	15	15	100.0	0.0	0.0	0.0	0.0
Tumors of the lung, pleura and thymus	Squamous-cell carcinoma of the lung	250	192	79.2	2.6	8.9	9.4	20.8
	Large-cell carcinoma of the lung	31	23	87.0	13.0	0.0	0.0	13.0
	Adenocarcinoma of the lung	250	192	79.2	2.6	8.9	9.4	20.8
	Bronchioloalveolar carcinoma	6	6	66.7	16.7	0.0	16.7	33.3
	Small-cell carcinoma of the lung	20	17	100.0	0.0	0.0	0.0	0.0
	Malignant mesothelioma	39	39	100.0	0.0	0.0	0.0	0.0
	Mesothelioma, other types	76	65	100.0	0.0	0.0	0.0	0.0
	Thymoma	29	27	100.0	0.0	0.0	0.0	0.0
Tumors of the female genital tract	Squamous cell carcinoma of the vagina	48	37	100.0	0.0	0.0	0.0	0.0
	Squamous cell carcinoma of the vulva	50	33	100.0	0.0	0.0	0.0	0.0
	Squamous cell carcinoma of the cervix	50	38	97.4	2.6	0.0	0.0	2.6
	Adenocarcinoma of the cervix uteri	50	36	69.4	13.9	2.8	13.9	30.6
	Endometrioid endometrial carcinoma	236	210	69.0	11.9	14.3	4.8	31.0
	Endometrial serous carcinoma	82	58	89.7	8.6	1.7	0.0	10.3
	Endometrial MMMT	28	24	91.7	4.2	0.0	4.2	8.3
	Endometrial carcinoma, low differentiated G3	13	11	90.9	9.1	0.0	0.0	9.1
	Endometrial clear cell carcinoma	8	4	100.0	0.0	0.0	0.0	0.0
	Endometrial stromal sarcoma	12	12	100.0	0.0	0.0	0.0	0.0
	Endometrioid carcinoma of the ovary	37	26	96.2	3.8	0.0	0.0	3.8
	Serous carcinoma of the ovary	50	41	97.6	0.0	2.4	0.0	2.4
	Mucinous carcinoma of the ovary	26	22	54.5	4.5	4.5	36.4	45.5
	Brenner tumor	9	7	100.0	0.0	0.0	0.0	0.0
Tumors of the breast	Invasive breast carcinoma of no special type	1311	854	97.7	0.2	2.0	0.1	2.3
	Lobular carcinoma of the breast	214	131	99.2	0.0	0.8	0.0	0.8
	Medullary carcinoma of the breast	26	22	100.0	0.0	0.0	0.0	0.0
	Tubular carcinoma of the breast	27	15	100.0	0.0	0.0	0.0	0.0
	Mucinous carcinoma of the breast	58	39	97.4	0.0	2.6	0.0	2.6
	Phyllodes tumor of the breast	50	36	100.0	0.0	0.0	0.0	0.0
Tumors of the digestive system	Adenomatous polyp, low-grade dysplasia	50	22	36.4	18.2	13.6	31.8	63.6
	Adenomatous polyp, high-grade dysplasia	50	30	40.0	16.7	10.0	33.3	60.0
	Adenocarcinoma of the colon	50	36	83.3	0.0	8.3	8.3	16.7
	Adenocarcinoma of the small intestine	10	6	66.7	0.0	0.0	33.3	33.3
	Gastric adenocarcinoma, diffuse type	146	113	55.8	8.0	8.8	27.4	44.2
	Gastric adenocarcinoma, intestinal type	144	115	60.9	16.5	5.2	17.4	39.1
	Gastric adenocarcinoma, mixed type	62	54	64.8	9.3	5.6	20.4	35.2
	Adenocarcinoma of the esophagus	50	39	28.2	23.1	17.9	30.8	71.8
	Squamous cell carcinoma of the esophagus	49	31	96.8	0.0	0.0	3.2	3.2
	Squamous cell carcinoma of the anal canal	50	26	92.3	0.0	0.0	7.7	7.7
	Cholangiocarcinoma	120	100	79.0	1.0	7.0	13.0	21.0
	Hepatocellular carcinoma	50	48	100.0	0.0	0.0	0.0	0.0
	Ductal adenocarcinoma of the pancreas	50	33	36.4	24.2	6.1	33.3	63.6
	Pancreatic/Ampullary adenocarcinoma	30	17	58.8	11.8	5.9	23.5	41.2
	GIST	50	34	100.0	0.0	0.0	0.0	0.0
Tumors of the urinary system	Noninvasive papillary urothelial carcinoma, pTa G2 low grade	177	177	94.9	2.3	1.7	1.1	5.1
	Noninvasive papillary urothelial carcinoma, pTa G2 high grade	141	141	91.5	3.5	4.3	0.7	8.5
	Noninvasive papillary urothelial carcinoma, pTa G3	187	187	92.5	2.1	5.3	0.0	7.5
	Urothelial carcinoma, pT2 to 4 G3	940	727	93.9	2.3	2.9	0.8	6.1
	Small-cell neuroendocrine carcinoma of the bladder	18	18	88.9	0.0	11.1	0.0	11.1
	Sarcomatoid urothelial carcinoma	25	25	100.0	0.0	0.0	0.0	0.0
	Clear cell renal cell carcinoma	858	507	100.0	0.0	0.0	0.0	0.0
	Papillary renal cell carcinoma	255	152	100.0	0.0	0.0	0.0	0.0
	Clear cell (tubulo) papillary renal cell carcinoma	21	10	100.0	0.0	0.0	0.0	0.0
	Chromophobe renal cell carcinoma	131	94	100.0	0.0	0.0	0.0	0.0
	Oncocytoma	177	109	100.0	0.0	0.0	0.0	0.0
Tumors of the male genital organs	Adenocarcinoma of the prostate	49	46	100.0	0.0	0.0	0.0	0.0
	Adenocarcinoma of the prostate (recurrence)	330	182	97.8	1.1	1.1	0.0	2.2
	Small-cell neuroendocrine carcinoma of the prostate	17	14	100.0	0.0	0.0	0.0	0.0
	Seminoma	50	50	100.0	0.0	0.0	0.0	0.0
	Embryonal carcinoma of the testis	50	44	100.0	0.0	0.0	0.0	0.0
	Yolk sack tumor	50	35	94.3	0.0	2.9	2.9	5.7
	Teratoma	50	24	95.8	4.2	0.0	0.0	4.2
Tumors of endocrine organs	Adenoma of the thyroid gland	114	106	100.0	0.0	0.0	0.0	0.0
	Papillary thyroid carcinoma	392	359	100.0	0.0	0.0	0.0	0.0
	Follicular thyroid carcinoma	158	148	100.0	0.0	0.0	0.0	0.0
	Medullary thyroid carcinoma	107	83	98.8	0.0	1.2	0.0	1.2
	Anaplastic thyroid carcinoma	45	37	100.0	0.0	0.0	0.0	0.0
	Adrenal cortical adenoma	50	49	100.0	0.0	0.0	0.0	0.0
	Adrenal cortical carcinoma	26	14	100.0	0.0	0.0	0.0	0.0
	Phaeochromocytoma	50	40	100.0	0.0	0.0	0.0	0.0
	Appendix, NET	22	16	93.8	0.0	6.3	0.0	6.3
	Ileum, NET	49	49	100.0	0.0	0.0	0.0	0.0
	Lung, NET	19	19	100.0	0.0	0.0	0.0	0.0
	Pancreas, NET	102	85	96.5	0.0	2.4	1.2	3.5
	Colorectal, NET	10	10	100.0	0.0	0.0	0.0	0.0
	gallbladder, NET	4	4	100.0	0.0	0.0	0.0	0.0
	Pancreas, NEC	13	11	100.0	0.0	0.0	0.0	0.0
	Colorectal, NEC	11	11	90.9	0.0	0.0	9.1	9.1
Tumors of haemotopoetic and lymphoid tissues	Hodgkin lymphoma	45	43	100.0	0.0	0.0	0.0	0.0
	Non-Hodgkin lymphoma	48	41	100.0	0.0	0.0	0.0	0.0
Tumors of soft tissue and bone	Tenosynovial giant cell tumor	45	43	100.0	0.0	0.0	0.0	0.0
	Leiomyoma	50	44	100.0	0.0	0.0	0.0	0.0
	Leiomyosarcoma	87	83	100.0	0.0	0.0	0.0	0.0
	Liposarcoma	132	117	100.0	0.0	0.0	0.0	0.0
	Angiosarcoma	73	64	98.4	0.0	1.6	0.0	1.6
	Angiomyolipoma	91	91	100.0	0.0	0.0	0.0	0.0
	Dermatofibrosarcoma protuberans	21	21	100.0	0.0	0.0	0.0	0.0
	Ganglioneuroma	14	14	100.0	0.0	0.0	0.0	0.0
	Granular cell tumor	53	48	100.0	0.0	0.0	0.0	0.0
	Kaposi sarcoma	8	8	100.0	0.0	0.0	0.0	0.0
	MPNST	13	13	100.0	0.0	0.0	0.0	0.0
	Myofibrosarcoma	26	26	100.0	0.0	0.0	0.0	0.0
	Neurofibroma	117	117	100.0	0.0	0.0	0.0	0.0
	Sarcoma, NOS	75	75	100.0	0.0	0.0	0.0	0.0
	Paraganglioma	41	41	100.0	0.0	0.0	0.0	0.0
	PNET	23	23	100.0	0.0	0.0	0.0	0.0
	Rhabdomyosarcoma	7	7	100.0	0.0	0.0	0.0	0.0
	Schwannoma	121	121	100.0	0.0	0.0	0.0	0.0
	Synovial sarcoma	12	12	100.0	0.0	0.0	0.0	0.0
	Osteosarcoma	43	27	100.0	0.0	0.0	0.0	0.0
	Osteosarcoma	39	33	100.0	0.0	0.0	0.0	0.0

Abbreviations: MUC5AC, mucin 5AC; MMMT, malignant mixed Müllerian tumor; GIST, gastrointestinal stromal tumor; NET, neuroendocrine tumor; NEC, neuroendocrine carcinoma; MPNST, malignant peripheral nerve sheath tumor; NOS, not otherwise specified; PNET, primitive neuroectodermal tumor.

## Discussion

Our comprehensive analysis of >100 tumor entities identified 44 tumor types and subtypes that at least occasionally express MUC5AC. The highest rates of positivity were seen for colonic adenomas (62%), adenocarcinomas of the esophagus (72%), pancreas (64%) and the stomach (44%) as well for mucinous carcinoma of the ovary (46%). Most other cancer types with glandular differentiation, such as endometrium cancer, adenocarcinoma of the cervix uteri, adenocarcinoma of the lung and cholangiocarcinoma were also MUC5AC positive in a fraction of cases. In principle, these findings are concordant with the literature, as several earlier reports have already described most of these tumor entities to express MUC5AC.^9,10,13,14,19,23,25,28,32–37^ However, the large number of analyzed tumor entities in our study allowed us to identify 22 tumor categories that can express MUC5AC but were never described to do so earlier. These included for example small-cell carcinomas of the urinary bladder, neuroendocrine tumor of the pancreas, appendix and colon as well as squamous cell carcinomas of the anal canal and the larynx. MUC5AC expression was also seen in 1 case each of angiosarcoma and medullary thyroid carcinoma.

The vast majority of MUC5AC positive cancers were derived from organs where at least some cells normally express MUC5AC. This particularly applies to carcinomas arising from the stomach, lung, and cervix uteri, where the physiologic expression of MUC5AC is well known.^2,8,12^ MUC5AC expression is also seen in few scattered epithelial cells of the normal colon as well as in columnar cells that can cover the anal transitional epithelium and the urothelium. It is possible that the same mechanisms that drive MUC5AC expression in these specialized cells also apply for MUC5AC expression in some tumors derived from these organs. It is of note that—irrespective of a cancer origin—patterns of MUC5AC expression often contain a limited number of strongly positive cells being interspersed between negative cancer cells in a fairly regular way (mosaic pattern). MUC5AC expression is also seen in carcinomas arising from tissues that completely lack positive MUC5AC immunostaining under physiological circumstances. In these tissues, MUC5AC expression may serve as a useful surrogate marker of neoplastic transformation. For example, the nonneoplastic pancreas does not express MUC5AC, while MUC5AC expression can be detected in most pancreatic adenocarcinomas and even in its precursor lesions as early as low-grade PanINs.^38^ The same applies to ovarian mucinous carcinomas.^28^ Studies have shown that the putative underlying mechanisms of MUC5AC neo-expression include promotor hypomethylation.^11^ MUC5AC transcription is also directly and indirectly regulated by various cancer-associated transcription factors (eg AP-1, SP-1, NF-kB, HNF-5a-alpha) and pathways (eg MAPK, Akt/PI3K)^39–41^ as well as by several mediators of inflammation (IL-1-beta, IL-6, TNF-alpha, IL-13).^41–44^

**Figure 4.**
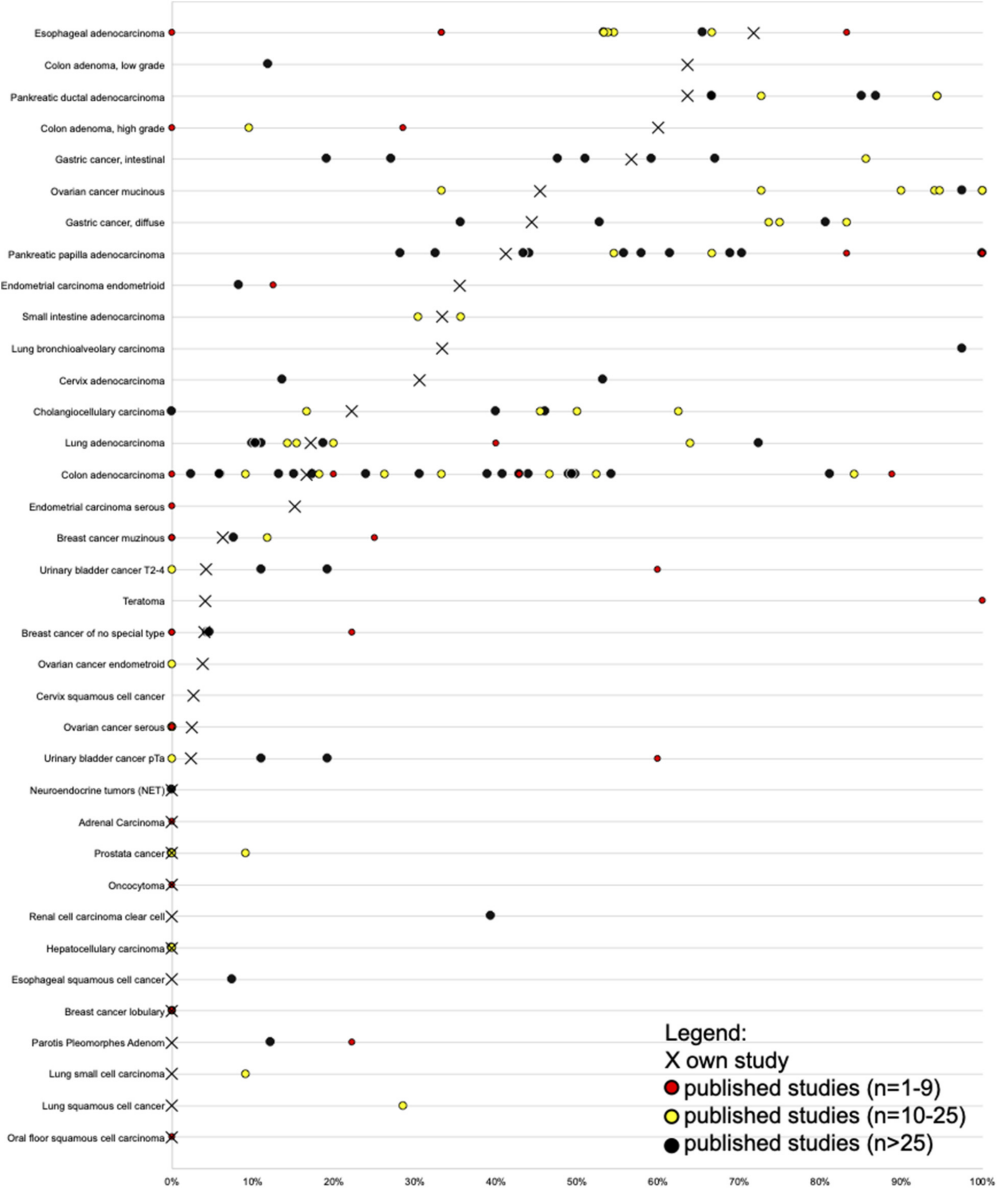
Graphical comparison of Mucin 5AC (MUC5AC) data from this study (x) in comparison with the previous literature. Red dots are used for studies involving 1 to 9 cases, yellow dots are used for studies involving 10 to 25 cases, and black dots are used for studies involving >25 cases.

Tumor types that are always MUC5AC negative are of diagnostic interest, because a positive MUC5AC immunostaining will virtually exclude such entities from diagnostic considerations. In the present analysis, 67 of 111 (60%) analyzed tumor types and subtypes did never show MUC5AC immunostaining. These tumor categories are the least likely to be the cause of a MUC5AC positive metastasis. Clinically relevant tumors with complete or almost complete absence of MUC5AC staining included small cell carcinoma of the lung (0% of 17), papillary thyroid carcinoma (0% of 359), breast cancer (2% of 1097), prostate cancer (2% of 228), soft tissue tumors (0.1% of 968), hematological neoplasias (0% of 111), and clear cell renal cell carcinoma (0% of 507). That the only earlier study analyzing MUC5AC by IHC in clear cell renal cell carcinoma had identified 39% positive cases^45^ illustrates the generally low concordance rate between IHC studies performed in different laboratories. A summary of published IHC data on MUC5AC expression is depicted in Figure 4 for all cancer types.

Our highly standardized analysis of 111 human tumor types and subtypes resulted in a ranking order according to the prevalence of MUC5AC expression (Figure 3). These data also show that most cancer types with infrequent MUC5AC positivity were considered “low expressors.” As in medullary thyroid carcinoma (Figure 2F), this tumor often shows few scattered MUC5AC (highly) positive cancer cells in an otherwise clearly negative tumor. Importantly, all absolute numbers and prevalences described in this study are specific to the reagents and the protocol used in our laboratory. It appears certain that the discrepant results between different studies describing MUC5AC IHC data are mainly due to the use of different commercially available antibodies, staining protocols and criteria for interpretation of staining. It is well known that different antibodies designed for the same target protein will vary markedly in their binding properties.^46–53^ This also applies to very common and important targets. For example, we have earlier shown the impact of different antibodies targeting epidermal growth factor receptor (EGFR) gene on the outcome EGFR analysis across human tumor types.^54^ Moreover, PD-L1 expression is routinely tested by IHC in cancers that are being considered for immune checkpoint-inhibitor therapy. The utilized PD-L1 antibodies are highly characterized but vary considerably in their binding properties. This results in a significant staining variability for PD-L1 antibodies both within tumor cells and nonneoplastic immune and stroma cells.^55^

That MUC5AC is broadly expressed across different tumor entities limits its use in the distinction of cancers of different origins. The occurrence of relevant subgroups of MUC5AC positive and negative tumors within many clinically relevant cancer types raises, however, the question of a possible prognostic or predictive role of MUC5AC expression. Several studies have indeed suggested a possible link of MUC5AC positivity to patient outcome in clear cell renal cell cancer,^45^ colorectal cancer,^14^ adenocarcinoma of the cervix,^31^ intrahepatic cholangiocarcinoma,^56^ gastric cancer,^32^ and nonsmall-cell lung cancer.^57^ Other established or proposed diagnostic applications include the distinction of serrated adenomas from hyperplastic polyps in the colon,^22^ the classification of gastric polyps and Barrett metaplasia^20^ as well as a parameter for malignancy in pancreatic biopsies.^19^ Moreover, high serum levels of MUC5AC have earlier been reported for patients with MUC5AC positive pancreatic adenocarcinomas, colorectal cancer, cholangiocarcinoma, gastric cancer, and biliary tract cancer.^58–63^ Serum MUC5AC measurement of patients with MUC5AC positive cancers could be utilized for detection of recurrence and measuring response to therapy. A limitation of the study could be the missing evaluability of about 22% of the tumor samples. However, a statistical bias, which could potentially result from the exclusion of noninterpretable samples, is highly unlikely in our study as noninterpretable samples were evenly distributed across all pathological diagnoses.

## Conclusions

In summary, our data provide a systematic overview of MUC5AC expression in human cancers using a standardized approach across all tissues and demonstrate potential diagnostic applications. Given a positivity rate of >60% in pancreatic cancer and a complete absence of MUC5AC in normal pancreatic tissue, MUC5AC IHC may be most useful for supporting a diagnosis of pancreatic cancer.
